# The Luminescent Inhomogeneity and the Distribution of Zinc Vacancy-Related Acceptor-Like Defects in N-Doped ZnO Microrods

**DOI:** 10.1186/s11671-016-1736-7

**Published:** 2016-11-22

**Authors:** Zhengrong Yao, Kun Tang, Zhonghua Xu, Jiandong Ye, Shunming Zhu, Shulin Gu

**Affiliations:** 1 School of Electronic Science and Engineering, Nanjing University, Nanjing, 210023 China; 2 School of Science, China Pharmaceutical University, Nanjing, 211198 China

**Keywords:** Zinc oxide, Microrod, Chemical vapor transport, Defect identification and distribution, Spatially resolved cathodoluminescence

## Abstract

Vertically aligned N-doped ZnO microrods with a hexagonal symmetry were fabricated via the chemical vapor transport with abundant N_2_O as both O and N precursors. We have demonstrated the suppression of the zinc interstitial-related shallow donor defects and have identified the zinc vacancy-related shallow and deep acceptor states by temperature variable photoluminescence in O-rich growth environment. Through spatially resolved cathodoluminescence spectra, we found the luminescent inhomogeneity in the sample with a core-shell structure. The deep acceptor-isolated V_Zn_ and the shallow acceptor V_Zn_-related complex or clusters mainly distribute in the shell region.

## Background

It is well-known that efficient p-type doping in ZnO thin films is extremely hard partially due to the very limited dopant solubility in high-quality lattice [1, 2]. Dopants are found to distribute along grain boundaries possibly due to the relatively degraded crystalline quality there. Grain boundaries would form low-resistive electrical channels, and the carrier transport is thus dominated by them, which has no practical significance [3]. However, micro-/nanostructured ZnO materials may provide an alternative to realize considerable doping efficiency by utilizing abundant surface areas. Similar as grain boundaries in thin films, surface areas in micro-/nanostructured ZnO are more chemically active, which results in lowered formation energies for impurities or defects. Therefore, doping is thought to be much easier on surfaces.

However, owing to such a different impurity or defect incorporation ability between bulk and surface, a spatially inhomogeneous distribution of the impurities or defects will naturally occur in ZnO micro-/nanostructures. Several groups have reported the inhomogeneity in terms of luminescent characterizations. Shalish et al. [4] have found that the luminescent intensity ratio between the below bandgap (surface) and the near band edge (NBE) emissions in ZnO nanowires depends on the wire radius. Pan et al. [5] have observed an increased intensity of deep-level (DL) emission when the surface aspect ratio becomes higher in their tapered ZnO nanorod. Foley et al. [6] have observed that the cathodoluminescence (CL) characteristics vary dramatically between the tip and sidewall of the ZnO nanorod as well as CL excitation depth. They also suggested that the green band (GB) luminescence originating from the ZnO surface is likely caused by oxygen vacancy (V_O_). Khranovskyy et al. [7] have further revealed that the NBE emission occurs primarily from the top (0001) planes of ZnO microrods (MRs) while the point defect V_O_-related visible emission mainly occurs from the side facets. Liao and Zhang [8] and Kaftelen et al. [9] have both reported that the ZnO nanostructures have a core-shell-structured luminescent distribution, and the visible luminescence is mainly emitted from the surface region where large numbers of V_O_ exist. However, Fabbri et al. [10] unambiguously gave the first evidence that zinc vacancy (V_Zn_) at the (10-10) non-polar surfaces is responsible for the GB luminescence of ZnO nanostructures by performing an exhaustive comparison between CL spectra and imaging and ab initio simulations.

All these prior literatures have demonstrated the luminescent inhomogeneity in ZnO micro-/nanostructures and that spatially resolved (SR) CL is a powerful tool to investigate this issue. However, the correlation between the DL (or GB) and the NBE luminescence and the responsible defects have not yet been fully established, leading to highly controversial attributions. Furthermore, none of the previous works has given information on the distribution of shallow acceptors in ZnO micro-/nanostructures, which is of crucial importance for further design and optimization of ZnO-based nanostructure devices. As a result, in order to explore the correlation between the luminescence and defects and the distribution of defects in the nanostructured ZnO material, we have employed Raman spectra, electrical characterization, low-temperature SR-CL, and temperature-dependent (TD) photoluminescence (PL) on our N-doped ZnO MR array samples. The experimental results have demonstrated the inhomogeneous luminescent distribution, and it was found out that the native defect-isolated V_Zn_ [11, 12] connected with the GB emissions and the shallow acceptors originating from V_Zn_-related complexes [13, 14] or clusters [15, 16] are mainly located at the shell region of the MRs.

## Methods

Vertically aligned N-doped ZnO MR array samples were homo-epitaxially grown on a high-quality ZnO template via chemical vapor transport method without any catalyst. The carrier gas was 100 SCCM N_2_, and 8 SCCM N_2_O was employed as both O and N precursors. A radio frequency plasma generator was used to ionize N_2_O to produce efficient O and N atoms. The detail of procedure and conditions can be referenced elsewhere [12].

The morphologies of the as-grown samples were characterized by JEOL JSM-7000F scanning electron microscopy (SEM), attached with a Gatan MonoCL system. The chemical configuration of the elements was determined by X-ray photoelectron spectrometry (XPS) with an Al K*α* X-ray monochromatic source at 1486.6 eV. Ar ion etching was performed in order to avoid the influence of surface absorption in the atmosphere, and the binding energy was calibrated by the C 1s peak at 285 eV. High-resolution X-ray diffraction (XRD) spectra were recorded to study the crystallinity of the samples on a Philips X’pert Pro diffractometer. The vibrational properties of the samples were investigated by a JOBIN YVON HR800 Raman system in the backscattering geometry with 514-nm radiation at room temperature. For analyzing the luminescence properties of a single MR, TD-PL excited by a He–Cd laser at the wavelength of 325 nm and SR-CL using variable acceleration voltage from 3 to 15 kV were carried out. The CL spectra and monochromatic imaging in this work were performed at the temperature of 100 K. To get the electrical parameters of N-doped ZnO MRs, back-gate field effect transistors (FETs) of a single MR were fabricated and measured.

## Results and Discussion

**Fig. 1:**
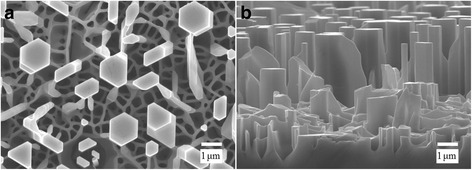
The **a** top view and **b** side-view SEM images of the vertically aligned N-doped ZnO MRs

Figure 1 shows the morphologies of the as-grown N-doped ZnO MR array. The images from top view and side view show an array of vertically well-aligned MRs with a hexagonal symmetry, which are parallel to each other. The in-plane scale of most MRs is around 1 μm while the height is around several microns. It is clear that there is a “foam-like” network of nanowalls below the MRs, which is entirely consistent with the experimental results reported previously [17, 18]. Since the metal catalyst was not used, the growth mechanism of the ZnO MRs is different from the traditional vapor–liquid–solid (VLS) mechanism and apparently via vapor–solid mechanism or the self-catalyzed VLS mechanism as proposed by Hu et al. [19]. When the growth process was over, the furnace was cooled down naturally to room temperature. However, the carbothermal reduction reaction did not stop immediately at the beginning of the cooling process due to the high furnace temperature. Therefore, the reaction of small amount of residual Zn and O atoms would form a layer of hexagonal head on the top of some MRs, as observed in Fig. 1.

**Fig. 2:**
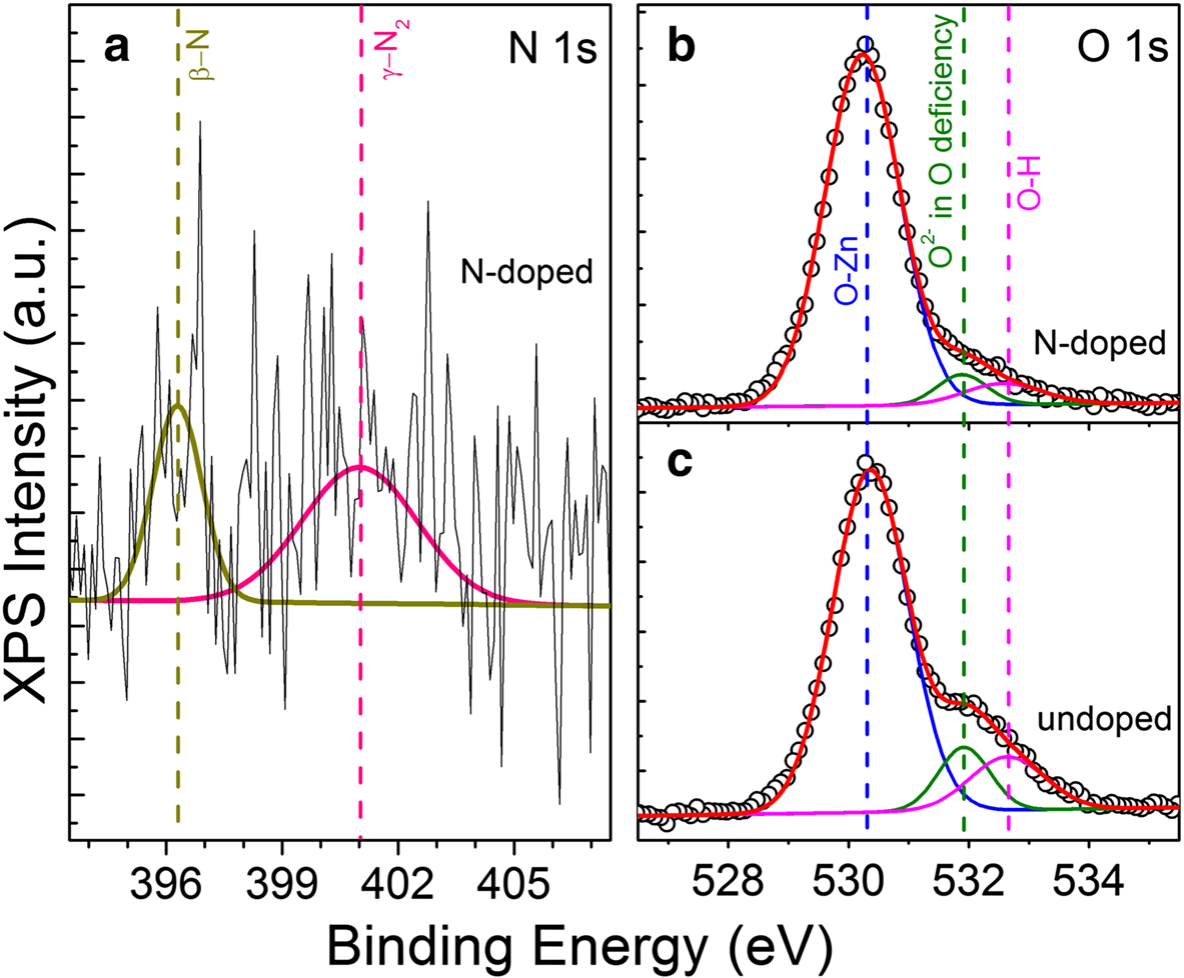
**a** The XPS N 1s spectrum of N-doped ZnO MRs. The XPS O 1s peaks of **b** N-doped and **c** undoped ZnO MRs

In order to investigate the chemical configuration of the elements in N-doped ZnO MRs, XPS was performed on the MR sample after Ar ion etching. As shown in Fig. 2a, we have observed two N 1s signals located around 396 and 401 eV, respectively, confirming the incorporation of N in our sample. Literatures show that the binding energy of the N 1s signal is very sensitive to the chemical environment. It has been reported that N can be incorporated into ZnO lattice in at least two chemical states, either as a N atom or a N_2_ molecule occupying an O site (N_O_ or (N_2_)_O_), corresponding to the peaks at 396.5 and 404.5 eV, respectively [20]. Wei et al. [21] have observed three features in the N 1s XPS spectra located at 396, 400, and 402 eV and have identified them as the N–Zn bond related to atomic N (β-N), well-screened and poorly screened molecular N (γ-N_2_), respectively. Therefore, we assigned the N 1s signals of 396 and 401 eV to β-N and γ-N_2_, respectively, indicating the formation of N_O_ and (N_2_)_O_ defects in our N-doped ZnO MRs.

The typical asymmetric O 1s peak of N-doped ZnO MRs is shown in Fig. 2b, which can be consistently fitted by three nearly Gaussian components, centered at 530.3, 531.9, and 532.6 eV, respectively. The main component on the low binding energy side can be attributed to the O–Zn bond, while the component with high binding energy is usually attributed to chemisorbed or dissociated OH or O species on the surface of ZnO material [22]. Then, according to the literature [23], the middle component is associated with O^2−^ ions in the O deficient regions within the ZnO lattice, and the intensity of this component may be partially connected with the concentration of V_O_, which has the lowest formation energy among the donor-like defects [24]. In addition, the XPS O 1s peak of undoped ZnO MRs grown with 1 SCCM high-purity O_2_ [12] is shown in Fig. 2c for comparison. It is obvious that the intensity of V_O_-related component in undoped sample is stronger than that of N-doped sample grown with higher VI/II ratio, indicating that V_O_ could be suppressed in O-rich growth environment to a great extent.

**Fig. 3:**
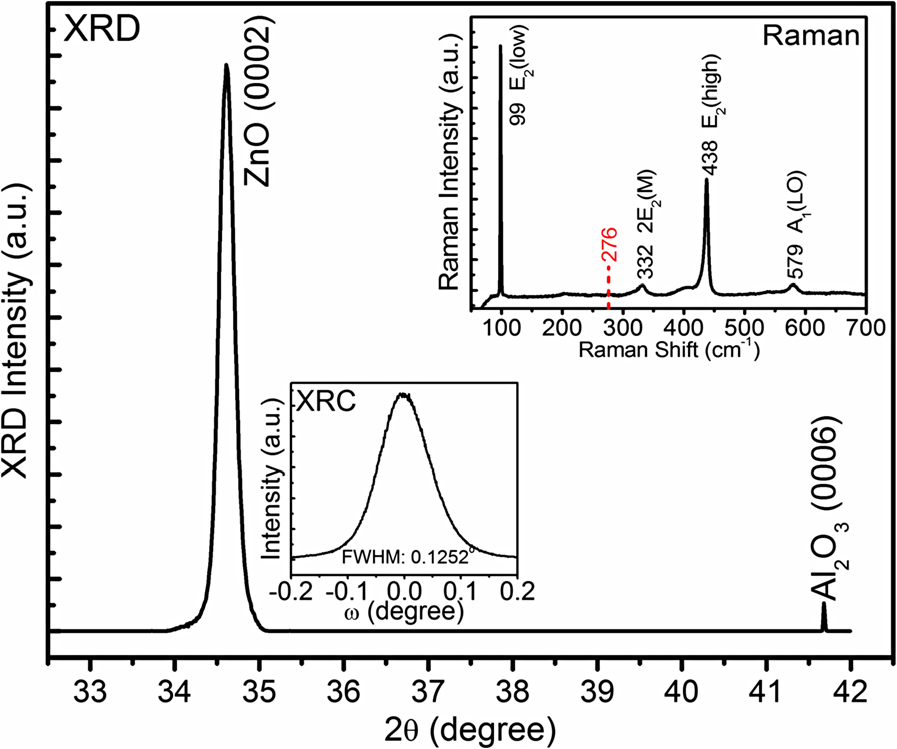
The XRD *ω*-2*θ* pattern of the N-doped ZnO MRs. The two *insets* depict the XRC of ZnO (0002) peak and the Raman spectra recorded at room temperature, respectively

The XRD and Raman characterizations have also demonstrated high crystalline quality for the sample. As seen from Fig. 3, only the ZnO (0002) in addition to the Al_2_O_3_ (0006) diffraction peaks can be observed, indicating that the ZnO MRs are oriented along the *c* axis with a well-ordered wurtzite structure. The full width at half maximum (FWHM) of the rocking curve (XRC) for the ZnO (0002) peak is 0.1252° as shown in the inset of Fig. 3, suggesting an excellent crystalline quality of the N-doped ZnO MRs. Raman spectrum (also shown in the inset of Fig. 3) shows classical ZnO lattice-related modes E_2_(low), 2E_2_(M), E_2_(high), and A_1_(longitudinal optical (LO)), located at 99, 332, 438, and 579 cm^−1^, respectively. The narrow width and high intensity of E_2_(low) and E_2_(high) are also an indication of high material quality. Moreover, in a previous paper, we observed additional modes (AMs) at 276, 510, 582, and 644 cm^-1^ in the Raman spectrum of N-doped ZnO MRs grown by 1 SCCM of N_2_O, while these AMs were absent in undoped ZnO MRs grown with high-purity O_2_ as O source [12]. It has been reported that the formation energy of native defect zinc interstitial (Zn_i_) increases in O-rich growth environment via a comprehensive first-principle investigation based on density functional theory [24]. Besides, Gluba et al. [25] reported that the AM at 274 cm^−1^ is related to small Zn_i_ clusters, which can be suppressed when the ZnO sample is deposited under O_2_ plasma. Lately, combined with defect formation energy calculations, Zhang et al. [26] demonstrated that Zn_i_ can be increased by the incorporation of N_O_ but suppressed by the presence of (N_2_)_O_. Consequently, considering higher O partial pressure in gas phase due to higher flow rate of N_2_O and the (N_2_)_O_ signal at 401 eV in the N 1s XPS spectrum, that no such AMs can be detected in the inset of Fig. 3 indicates a suppression of Zn_i_ small cluster-related native donors.

**Fig. 4:**
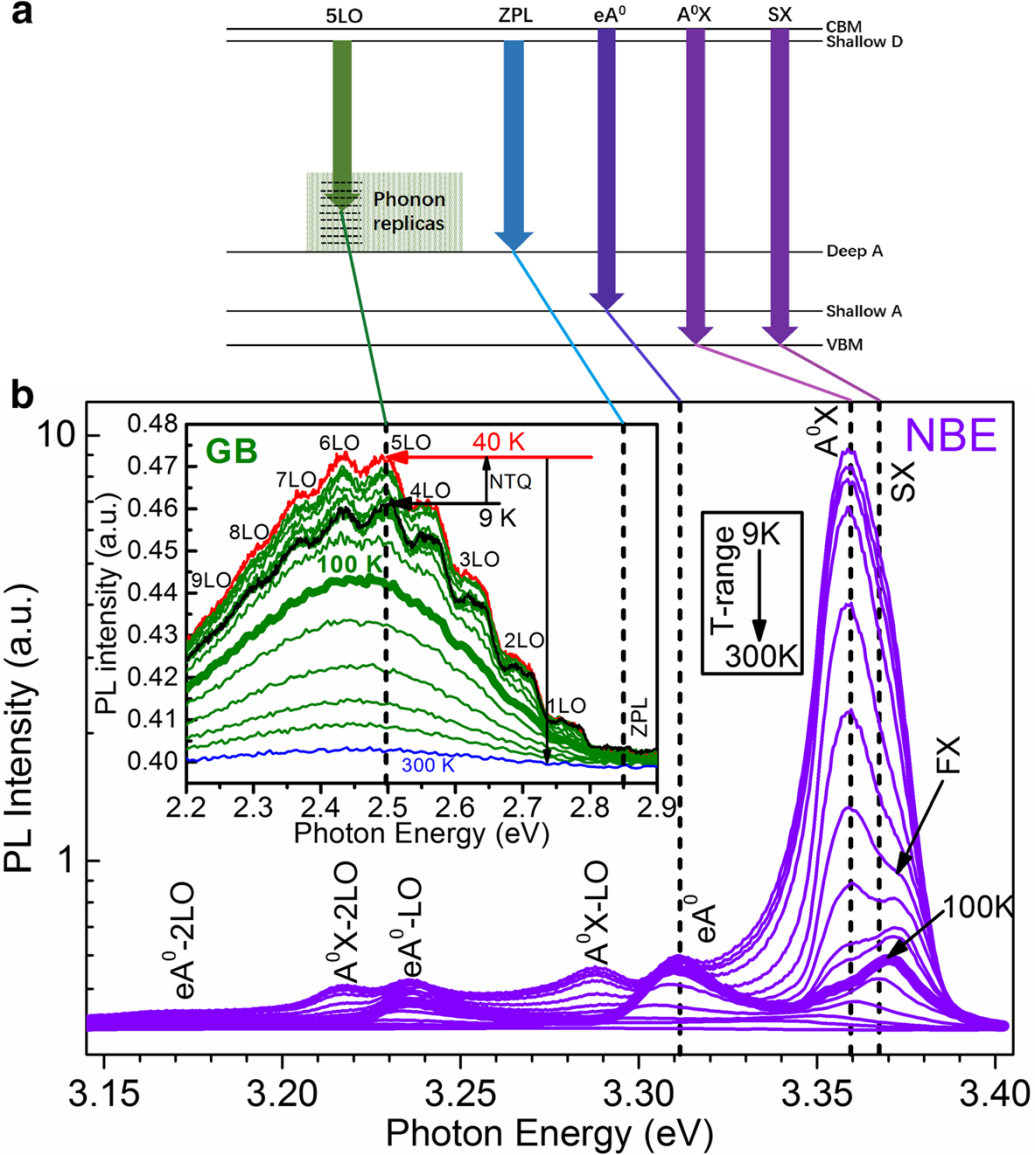
The TD-PL spectra of the N-doped ZnO MRs recorded from 9 to 300 K at the NBE region. The *inset* shows the corresponding GB emissions. The *top panel* draws a schematic energy band diagram and marks the origin of the GB, eA^0^, and A^0^X emissions, respectively

In ref. [12], we have discussed the optical properties of the undoped ZnO MRs in detail, and the NBE emission recorded at 9 K is dominated by excitons bound to neutral donors (D^0^X) and its two phonon replicas, located at 3.362, 3.291, and 3.218 eV, respectively. Provided the incorporation of N and the suppression of such a chief native donor Zn_i_, as shown in Fig. 4, the strongest NBE emission peak at 3.359 eV in the 9 K PL spectrum of N-doped ZnO MRs is assigned to excitons bound to neutral acceptors (A^0^X) [27–29]. Considering the free exciton (FX) line at 3.377 eV, the acceptor here is a shallow acceptor with its binding energy of around 18 meV. Due to the uncertainty of the Haynes factor for acceptors in ZnO, it is difficult to give the accurate energy level of this shallow acceptor. Taking the Haynes factor of 0.1–0.2, the energy level is around 90–180 meV [30]. Regarding the identification of the shallow acceptor in N-doped ZnO materials, there is no unified conclusion until now. The N_O_ was widely considered as a shallow acceptor for a long time. However, recent calculations demonstrated that the N_O_ has an exceedingly high ionization energy of 1.3 eV [31], supported by the experimental results of Huang et al. [32], which means N_O_ is not the natural candidate for explaining the observed A^0^X. Lately, more and more papers have a tendency to assign the unidentified shallow acceptor to a *native*-*extrinsic* complex or native defect *cluster* complex, including 2V_Zn_ − D_Zn_ (D = P, As, Sb) [33, 34], V_Zn_ − N_O_ [13], 2V_Zn_ − V_O_ [35], 3V_Zn_ − D_i_ (D = P, As, Sb) [36], and V_Zn_ clusters [16]. In fact, the successful demonstration of ZnO homo-junction-based light-emitting devices [37] and even laser diodes [38] undoubtedly supports the existence of shallow acceptor state(s) in group VA-doped ZnO materials. In our case, considering the chemicals incorporated, it is highly possible that the shallow acceptors in N-doped ZnO MRs be V_Zn_ − N_O_ or V_Zn_ clusters since N signals are detectable by XPS and energy-dispersive spectra [12].

In addition to the A^0^X and FX, a shoulder at 3.368 eV must be added for a better fitting of the NBE region. Regarding the assignment, it has been reported that surface exciton (SX) emission may occur in this spectral range, especially for ZnO nanostructures because of the large surface-volume ratio [39]. Moreover, the temperature dependence of the peak shows that the intensity decreases dramatically with increasing temperature, which is a typical behavior of SX [12]. Therefore, we attribute the shoulder at 3.368 eV to SX, which originates from the ionized bound excitons due to the surface depletion. For the attribution of the hump at 3.311 eV, according to our previous study and the temperature dependence of the peak position [12], we ascribe it to free electrons to acceptors (eA^0^). The acceptor state is thus around 120–130 meV, which is consistent with the acceptors responsible for A^0^X. The rest peaks at the lower energy side are ascribed to the first and second LO phonon replica of A^0^X or eA^0^, which have been denoted in Fig. 3 correspondingly. In addition, the GB emissions with zero-phonon line (ZPL) around 2.85 eV and its nine LO phonon replicas are presented in the inset of Fig. 4. It is found that the intensity of the GB increases first with increasing measuring temperature until 40 K, and this anomalous phenomenon is called negative thermal quenching effect which further ensures that the GB emissions in N-doped ZnO MRs originate from shallow donors to the isolated V_Zn_ with a relatively deep acceptor energy level [11, 12]. Note that the GB intensity (~0.47) is negligible as compared to that of the NBE (~10); it thus implies that shallow donors are suppressed to some extent, which is consistent with Raman spectrum.

**Fig. 5:**
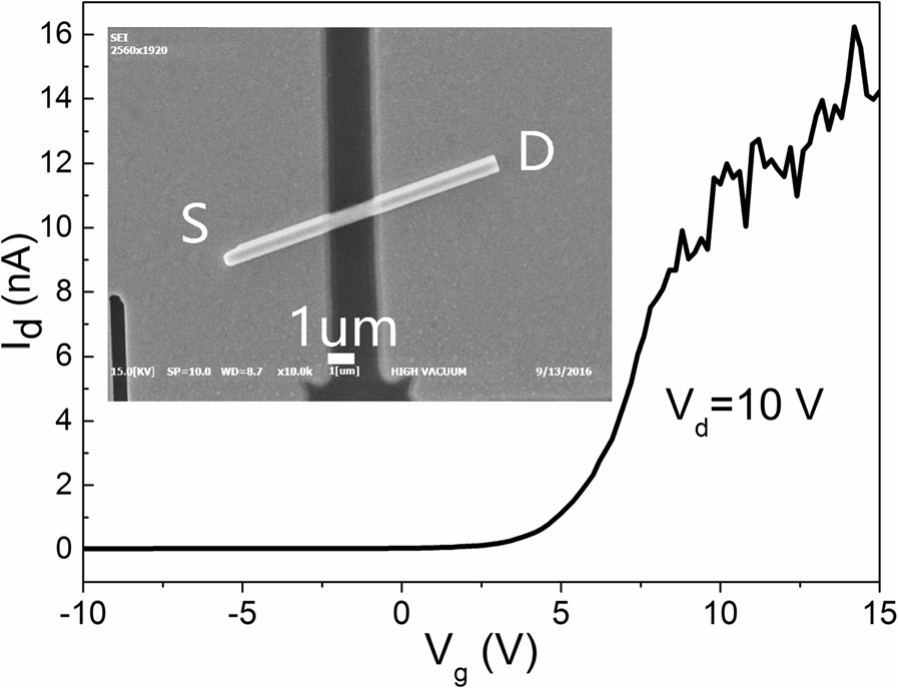
The I_d_–V_g_ curve of the N-doped ZnO MR FET under V_d_ = 10 V. The *inset* shows the SEM image of the FET

In order to obtain the electrical properties of the N-doped ZnO MRs, field-effect transistors (FETs) of individual MRs were fabricated by standard photolithography. N-doped ZnO MRs were first transferred to a p^+^-silicon wafer with a 200-nm thick silicon oxide on the surface, which served as the back-gate electrode of the transistor. Microcontact windows were defined on the ends of the MRs, and then, the electrodes with 10 nm of Ni followed by 150 nm of Au were formed by electron-beam evaporator and subsequent lift-off. The inset in Fig. 5 shows the SEM image of the N-doped ZnO MR FET, and the channel length between the electrodes is around 3 μm. Meanwhile, the drain current (I_d_) versus gate voltage (V_g_) curve under a drain voltage (V_d_) of 10 V is shown in Fig. 5. According to the literature [40], the carrier concentration (Q) in N-doped ZnO MR can be estimated by the following equation: *Q* = (*V*_th_/*e*)[2πε_r_ε_0_/ln(4h/d)]/(πd^2^/4), where *V*_th_, *e*, ε_r_, ε_0_, h, and d are the threshold voltage obtained from the transconductance in Fig. 5, the electron charge, the effective dielectric constant (3.9 for SiO_2_), the dielectric constant in vacuum, the thickness of gate oxide layer, and MR diameter (~1 μm), respectively. The carrier concentration is thus estimated to be ~2.3 × 10^15^ cm^−3^. The high resistance of the MR implies the existence of acceptors, which is consistent with the PL signatures of shallow and deep acceptors related to V_Zn_.

Utilizing the optical signatures of the native defects, one can easily establish the relation between native defects and the radiative emission. However, the spatial resolution for usual PL system is well above the micrometer range, which leads to averaging over many N-doped ZnO MRs on the sample surface. Although PL measurement on individual submicron structures is possible, the accuracy of the technique is limited by the diffraction limit and poor signal-to-noise ratio [6]. In order to gain insight on the local luminescence properties of a single N-doped ZnO MR and to reveal the distribution of native point defect, an approach is to measure the optical response via a luminescence spectroscopy with a scanning tunneling microscope (STM), which is acquired by retracting the STM tip from the sample surface for a few 100 nm [41, 42]. Another economic approach is to use the SR-CL spectra equipped within a regular SEM system [4–10], which is also of high spatial resolution due to the very tiny spot size of the electron beam. As a result, the SR-CL and monochromatic imaging were performed in a sample area of 6 × 4.8 μm^2^ with an acceleration voltage of 15 kV at 100 K.

**Fig. 6:**
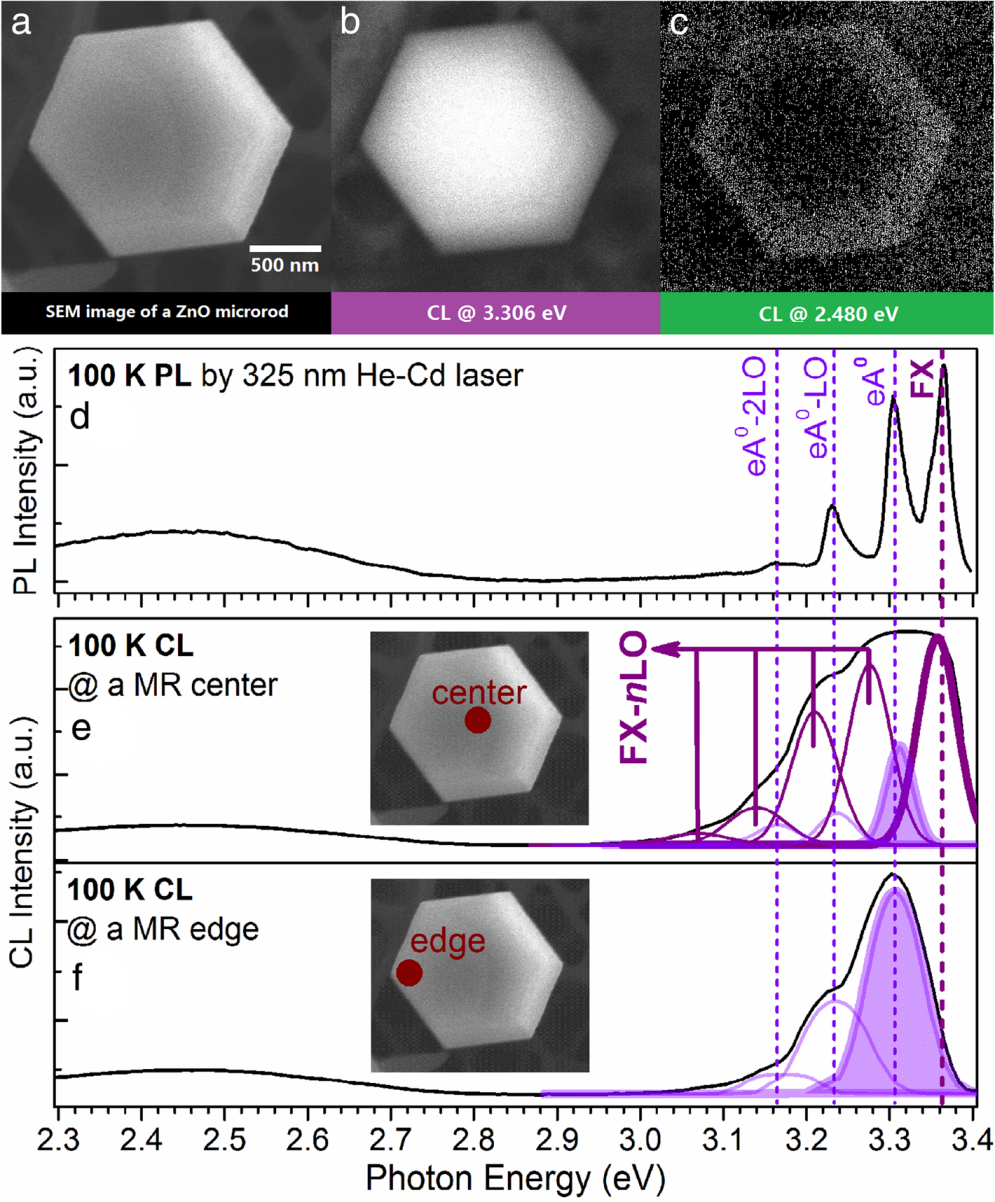
The SR-CL spectra and monochromatic images of a single N-doped ZnO MR performed with an accelerating voltage of 15 kV at 100 K. **a** The top view SEM image of the selected MR. **b** The CL monochromatic mapping of the MR taken at 3.306 eV. **c** The CL monochromatic mapping of the MR taken at 2.480 eV. **d** The PL spectrum measured at 100 K. **e** The CL spectrum obtained in spot mode from the center of the MR. **f** The CL spectrum obtained in spot mode from the edge of the MR

Figure 6a shows the top view SEM image of one single hexagonally shaped N-doped ZnO MR in the investigated area, while the CL monochromatic 2D mapping of this MR recorded at a photon energy of 3.306 eV (375 nm) and 2.480 eV (500 nm) are shown in Fig. 6b, c, respectively. It can be noticed that the ultraviolet luminescence is stronger at the center of the MR, but in contrast, the green band luminescence mainly originates from the edge within a thickness of around 200–300 nm. Taking the advantage of high spatial resolution of the CL technique, Fig. 6e, f presents two CL spectra obtained in spot mode from the center and edge, respectively. Figure 6d shows the 100 K PL spectrum for identification of the optical transitions in the CL spectra. By comparing the CL spectra with the PL spectrum, the 3.306 eV peak corresponds to the eA^0^ while the 2.480 eV peak corresponds to the GB. Owing to the width of CL spectra that are much larger than that of the PL, peak deconvolution process has been employed for the CL spectra according to the well-resolved PL peaks. As can be seen from the deconvoluted components, the intensified eA^0^ emission at the center observed in Fig. 6b actually originates from the contribution of FX and its first LO-phonon replica whereas the actual intensity of eA^0^ emission at the center is weaker than that at the edge. As the eA^0^ is linked to the shallow acceptors, the result indicates that more shallow acceptors are located along the edge of the MR. For the GB luminescence, the integrated intensity ratio of the GB/NBE is 0.179 and 0.342 for center and edge, respectively, indicating that more isolated V_Zn_ defects are located near the edge area, similar to the monochromatic observation from Fig. 6c.

**Fig. 7:**
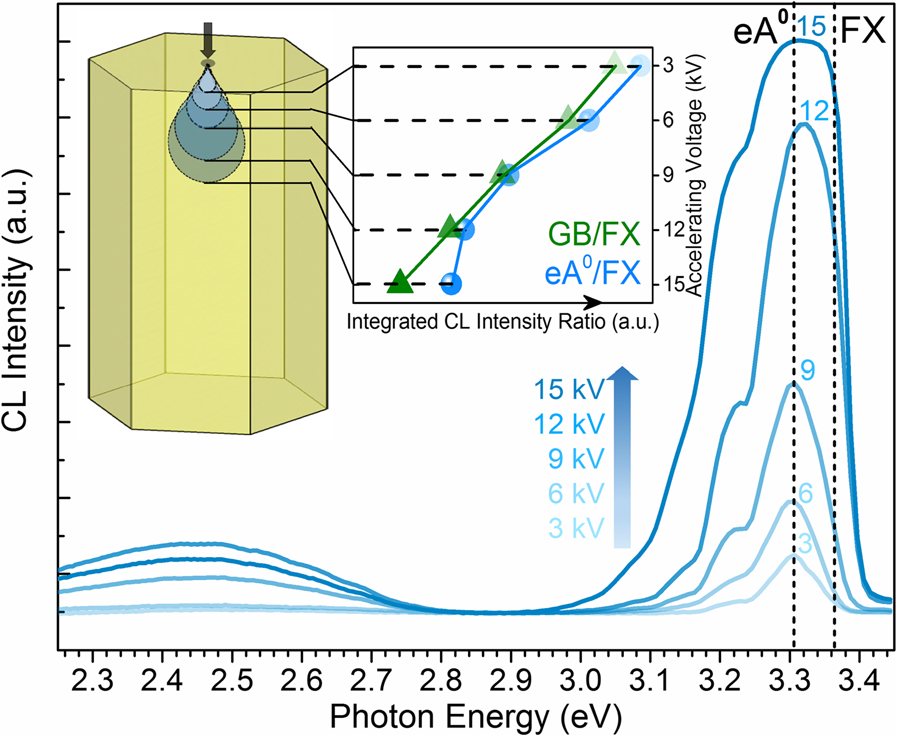
The CL spectra taken at 100 K in spot mode at the center of the N-doped ZnO MR with five different acceleration voltages of 3, 6, 9, 12, and 15 kV. The *inset* shows the integrated intensity ratio of eA^0^/FX and GB/FX as a function of the accelerating voltage (i.e., detection depth)

In fact, the inhomogeneous distribution of the acceptor-like defects occurs not only between the center and edge of the MRs but also between the top surface and the bulk underneath the surface. To attest this issue, depth-resolved CL measurement at 100 K has been carried out with various acceleration voltages at the center point. Figure 7 shows the CL spectra recorded at five different acceleration voltages of 3, 6, 9, 12, and 15 kV, which correspond to the respective average generation depths of 40, 110, 210, 340, and 500 nm in ZnO material, according to the calculation results through the Monte-Carlo simulation CASINO in literature [43]. As plotted in the inset of Fig. 7, the integrated intensity ratio of eA^0^/FX and GB/FX both decreases with increasing acceleration voltage due to the core of the N-doped ZnO MR has relatively less V_Zn_-related acceptor-like complexes.

**Fig. 8:**
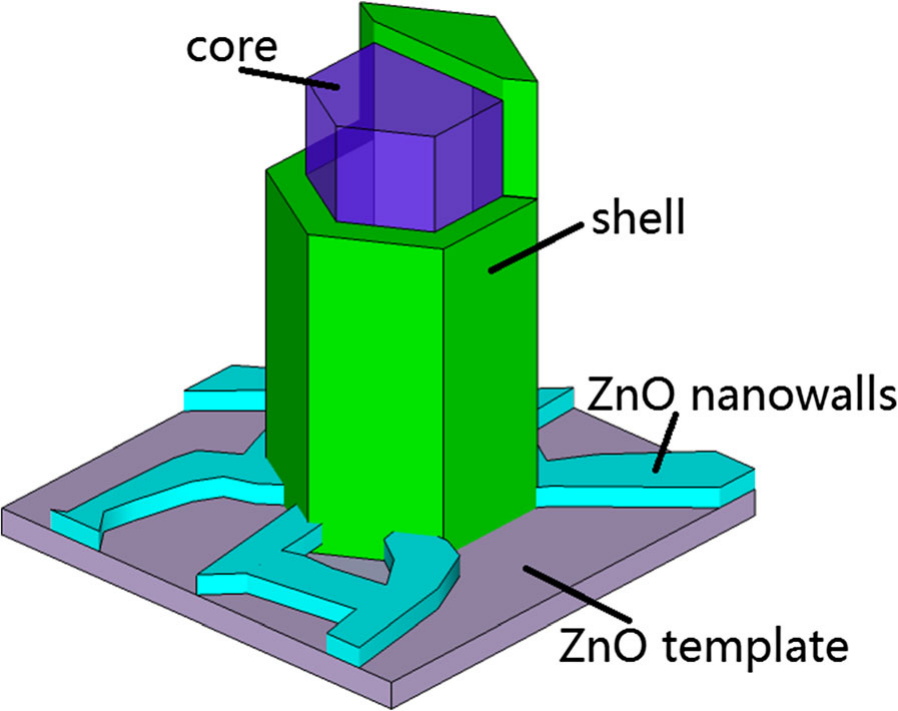
The schematic model of one N-doped ZnO MR with core-shell-structured luminescent inhomogeneity

The inhomogeneous distribution of the acceptor-like defects can be unified to the difference between surface and bulk. It is a well-known fact that surface defects of metal oxides function as adsorption sites [44]. The adsorption of a gas molecule, such as O_2_ or H_2_O, on the surface of ZnO will trap the free electrons to form negative O_2_^−^ or OH^−^ ions. Therefore, the adsorbates acting as acceptors deplete the surface electron states, leaving behind positively charged native defects or dopants near the surface, resulting in the space charge region and band bending of the surface [45, 46]. Due to larger surface-to-volume ratio, such a surface effect is more significant on ZnO nanostructures, compared with bulk materials. A layer that is depleted of mobile electrons is then created at the surface of the ZnO MR, the width of which is up to more than 100 nm [47]. Moreover, it has been reported that the V_Zn_^−^ centers only exist in the depletion layer [48], which could compensate for an intrinsic charge imbalance. In addition to the results reported by Fabbri et al. [10], all the above discussions suggest that both the V_Zn_-related complex shallow acceptors and the isolated V_Zn_ deep acceptors are mainly located near the surface region of the MRs. The intensified FX in bulk area and the disappearance of the peak at the surface area indicate that the crystalline quality is relatively better in bulk and relatively worse at surface. Possibly, for this very reason, more acceptor-like isolated and complex defects can form at the MR surface region. The “core-shell” luminescent structure of the N-doped ZnO MRs has been schematically drawn in Fig. 8. With further design and optimization of the experiment conditions, it might be possible to achieve a co-axial p–n junction which can be potentially applied to co-axial light-emitting devices.

## Conclusions

In conclusion, vertically aligned N-doped ZnO MRs with excellent crystalline quality were carried out via the chemical vapor transport method. The experimental results of Raman and TD-PL investigations demonstrate that the shallow donor-like defect Zn_i_ has been suppressed in O-rich growth environment, while on the other hand, N incorporation induced shallow acceptor state: V_Zn_-related complex or clusters are stable. Through comprehensive comparison between SR-CL spectra and monochromatic images taken at 100 K, we find the luminescent inhomogeneity of N-doped ZnO MRs, which has core-shell structure. The isolated V_Zn_ connected with the GB- and V_Zn_-related complexes or clusters associated with the eA^0^ emission mainly distributes in the shell of a thickness about 200–300 nm. The present study has provided a microscopic model for the distribution of acceptors in N-doped ZnO micro-/nanostructures and is certainly crucial for further design of one-dimensional ZnO nanostructure devices.

## Abbreviations

A^0^X: Excitons bound to neutral acceptor
CL: Cathodoluminescence
D^0^X: Excitons bound to neutral donor
DL: Deep-level
FET: Field-effect transitor
FWHM: Full width at half maximum
FX: Free exciton
GB: Green band
LO: Longitudinal optical
MR: Microrod
NBE: Near band edge
PL: Photoluminescence
SEM: Scanning electron microscope
SR: Spatially resolved
STM: Scanning tunneling microscope
SX: Surface exciton
TD: Temperature-dependent
VLS: Vapor–liquid–solid
V_O_: Oxygen vacancy
V_Zn_: Zinc vacancy
XPS: X-ray photoelectron spectrometry
XRD: X-ray diffraction
ZPL: zero-phonon line
